# A prospective study of the association between serum klotho and mortality among adults with rheumatoid arthritis in the USA

**DOI:** 10.1186/s13075-023-03137-0

**Published:** 2023-08-16

**Authors:** Qin-cheng Che, Qian Jia, Xiao-yu Zhang, Shu-ning Sun, Xiao-jie Zhang, Qiang Shu

**Affiliations:** 1 Department of Rheumatology, Qilu Hospital, Cheeloo College of Medicine, Shandong University, No.107, West Culture Road, Lixia District, Jinan, 250012 China; 2 Department of Rheumatology, Qilu Hospital, Shandong Provincial Clinical Research Center for Immune Diseases and Gout, Jinan, China

**Keywords:** Rheumatoid arthritis, Klotho, Mortality

## Abstract

**Background:**

While it is known that klotho has negative regulatory effects in a variety of diseases such as metabolic disorders and kidney disease, the specific role of klotho in rheumatoid arthritis (RA) and its effect on mortality are unclear. This study investigated the association between serum klotho levels and mortality in patients with RA.

**Methods:**

This study included 841 adults with RA from the National Health and Nutrition Examination Survey (NHANES) from 2007 to 2016 to extract the concentrations of serum klotho. The association between klotho and RA was determined using Cox regression, Kaplan–Meier (KM) curves, and restricted cubic spline (RCS) models.

**Results:**

A total of 841 patients with RA were included in this study, who were divided into four groups based on the quartiles of serum klotho levels (Q1, Q2, Q3, and Q4). Cox regression analysis with adjustment for covariates revealed that high levels of klotho lowered the risk of both all-cause and cardiovascular mortality compared to the Q1 group. The KM curve analysis suggested that this effect was more pronounced for all-cause mortality. The RCS-fitted Cox regression model indicated a U-shaped correlation between serum klotho levels and RA mortality. The risk of all-cause mortality increased with decreasing serum klotho levels below a threshold of 838.81 pg/mL. Subgroup analysis revealed that the protective effect of klotho was more pronounced in patients with the following characteristics: male, white ethnicity, age ≥ 60 years, body mass index < 25 kg/m^2^, estimated glomerular filtration rate ≥ 60 mL/ (min × 1.73 m^2^), and 25-hydroxyvitamin D level ≥ 50 nmol/L.

**Conclusion:**

Serum klotho levels had a U-shaped correlation with all-cause mortality in patients with RA, indicating that maintain a certain level of serum klotho could prevent premature death.

## Background

Rheumatoid arthritis (RA) is a chronic disease that manifests primarily as progressive joint damage and can lead to permanent disability and a higher mortality rate [1]. RA is also associated with a wide range of comorbidities such as osteoporosis, cardiovascular disease, and cancer [2]. As global RA incidence has increased annually [3], early intervention and targeted treatment are particularly important for improving patient survival [4, 5]. The emergence of multiple therapeutic targets for RA in recent years has been effective in slowing disease progression and reducing comorbidities. However, the mortality rate in patients with RA remains higher than that of the general population [6, 7]. Thus, there is a need to determine markers associated with RA mortality to facilitate the identification of at-risk populations for early intervention.

Klotho was first reported to be associated with aging phenotypes in 1997 [8]. Klotho proteins exist in two forms in the human body: membrane type and soluble type. Soluble klotho is secreted by cells or cleaved from membrane klotho. The soluble type of klotho can achieve biological functions through blood circulation [9], regulating target tissues by ameliorating oxidative stress, inhibiting inflammation, and modulating apoptosis [10].

Many previous studies have confirmed that klotho has negative regulatory effects and may be involved in a variety of conditions, such as altered cognitive function [11], metabolic disorders [12], kidney disease [13], and skeletal muscle reduction [14]. Serum klotho concentrations are significantly higher in patients with RA than in controls, and there is a significant association between klotho concentration and disease activity [15]. However, the specific role of klotho in RA and whether it affects the occurrence of mortality in RA patients are unclear.

In this study, we aimed to explore the association between serum klotho levels and RA-related mortality in adults. We extracted serum klotho data from the National Health and Nutrition Examination Survey (NHANES). The combined effects were analyzed using Cox regression models, restricted cubic spline (RCS) models, and Kaplan–Meier (KM) curves, with the aim of providing new insights into therapeutic interventions for RA and a basis for subsequent in-depth studies.

## Materials and methods

### Study population

The NHANES (https://www.cdc.gov/nchs/nhanes/) is a population-based cross-sectional survey that assesses the health and nutritional status of adults and children in the USA. A representative sample of approximately 10,000 individuals was randomly included in each 2-year study period. Data were primarily collected through interviews (e.g., demographic, socioeconomic, and health-related information) and examinations (e.g., medical examinations, physiological measurements, and laboratory tests) performed by trained medical personnel. NHANES was approved by the National Center for Health Statistics Institutional Review Board, with all participants providing written informed consent [16]. The findings help determine the prevalence of major diseases and risk factors for diseases, and provide data to support nutrition and health policy development.

**Fig. 1:**
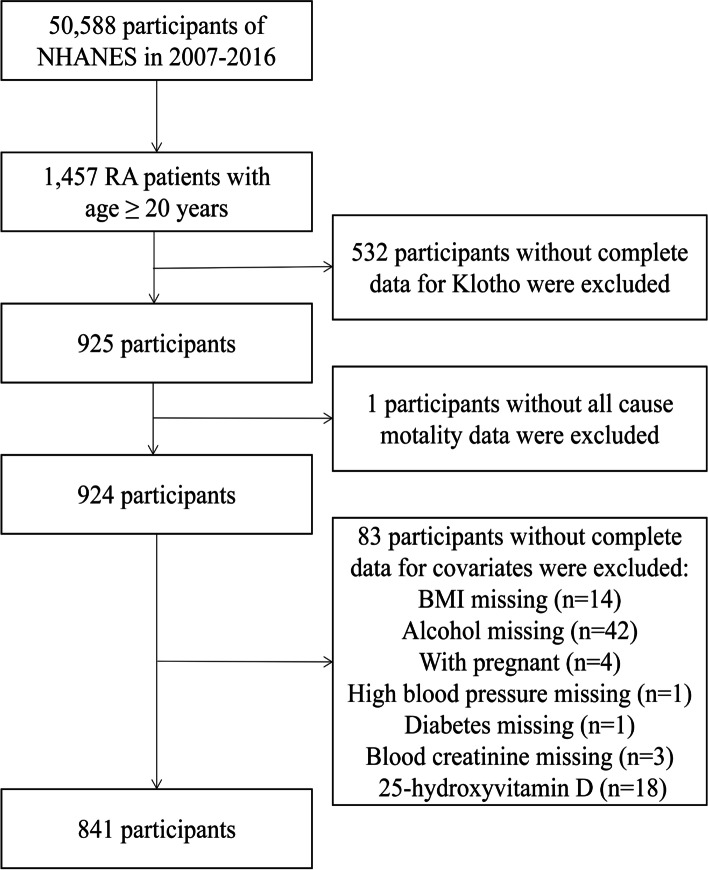
The flowchart of the study

In our study, we combined data from five cycles of NHANES between 2007 and 2016. We included all adults aged ≥ 20 years who had completed the arthritis survey (i.e., those selecting “rheumatoid arthritis” as the response to the following question: “which type of arthritis was it?”) and had undergone measurement of serum klotho. Participants with missing data on covariates were excluded. A total of 841 participants with RA were included in the analysis (Fig. 1).

### Measurement of serum klotho

According to the NHANES website, the klotho assay has been validated in previous studies. Serum samples were stored at − 80 °C until assay analysis. Klotho detection was performed using a commercially available enzyme-linked immunosorbent assay (ELISA) kit (IBL International, Tokyo, Japan). The samples were analyzed in duplicate, and the average of two values was used to calculate the final value. Klotho concentrations were also analyzed in duplicate in each ELISA plate, and quality control was performed accordingly. Serum klotho concentrations were divided into quartiles for statistical analysis: Q1 (< 25th percentile, 277–635 pg/mL), Q2 (25–50th percentile, 635–775 pg/mL), Q3 (50–75th percentile, 775–980 pg/mL), and Q4 (≥ 75th percentile, 980–3178 pg/mL); Q1 was used as the reference.

### Mortality outcome

Information on mortality status and follow-up time was collected through the National Death Index mortality database from NHANES (up to April 26, 2022). The cause of death was determined using the International Classification of Diseases 10th Revision, and the primary outcomes of our study were all-cause and cardiovascular mortality (I00–I09, I11, I13, I20–I51).

### Covariates

We included a number of factors that potentially affected the outcomes as covariates: sex (female, male), age, race (Non-Hispanic white, Non-Hispanic black, Mexican American, others), education level (less than 9th grade, 9–11th grade, high school graduate, some college or AA degree, college graduate or above), marital status (married or living with a partner; never married; widowed, divorced, or separated), family poverty income ratio (PIR, < 1.0, 1.0–3.0, or ≥ 3.0), drinking habit (< or ≥ 12 alcoholic drinks/lifetime), cotinine (reflects the smoking status and estimates environmental exposure to tobacco), hypertension (yes, no), diabetes (yes, no), body mass index (BMI) (< 25, 25–30, ≥ 30 kg/m^2^), physical activity (< 600, 600–1199, ≥ 1200 MET min per week), estimated glomerular filtration rate (eGFR) (estimated using the Chronic Kidney Disease Epidemiology Collaboration equation [17]), serum 25-hydroxyvitamin D (< 50, ≥ 50 nmol/L), and the study cycle (2007–2008, 2009–2010, 2011–2012, 2013–2014, 2015–2016). The serum cotinine concentration was natural logarithm-transformed as a continuous variable.

### Statistical analyses

Patients with RA were divided into four groups based on the quartiles of serum klotho concentrations (Q1, Q2, Q3, and Q4). The characteristics of the included population were analyzed using descriptive statistics. Serum cotinine concentrations were not normally distributed. Continuous variables were compared using analysis of variance or the Kruskal–Wallis *H*-test, and categorical variables were compared using the chi-square test. Appropriate sampling weights were considered in all analyses.

To assess the association of serum klotho concentration with all-cause and cardiovascular mortality, we used Cox proportional hazards regression analysis to calculate the corresponding hazard ratios (HRs) and 95% confidence intervals (CIs). Three models were established: model 1 (non-adjusted), model 2 (adjusted for sex, age, race, and study cycle), and model 3 (adjusted for sex, age, race, education level, marital status, PIR, cotinine, drinking, hypertension, diabetes, BMI, physical activity, eGFR, 25-hydroxyvitamin D, and study cycle). KM curves for all-cause and cardiovascular mortality were plotted separately for follow-up time (months) according to the Klotho grouping described above. Statistical differences between the groups were compared using the log-rank test.

An RCS model was established to investigate the nonlinear relationship between changes in klotho concentration and the risk of all-cause or cardiovascular mortality in patients with RA. This model was adjusted for sex, age, race, education level, marital status, PIR, cotinine, drinking, hypertension, diabetes, BMI, physical activity, eGFR, 25-hydroxyvitamin D level, and study cycle. If a nonlinear relationship was satisfied, the Cox proportional hazards regression model was fitted to determine whether there was a threshold point associated with the HR, as well as to verify the relationship between mortality and changes in klotho concentration on both sides of the threshold.

Klotho concentrations were divided into two groups: the low and the high groups, according to the thresholds determined via the RCS-fitted Cox regression. We compared the risk of all-cause mortality of the two groups and performed subgroup analysis with sex, age, ethnicity, BMI, eGFR, and 25-hydroxyvitamin D levels. We explored whether there was a significant interaction between 25-hydroxyvitamin D and klotho by testing whether the coefficients of the product terms or the relative excess risk due to the interaction were statistically significant using Cox regression.

All analyses in this study were conducted using R version 4.2.1. A two-sided *P*-value of < 0.05 was considered statistically significant.

## Results

### Patient characteristics

A total of 841 RA patients (57.79% were ≥ 60 years old, and 57.91% were female) were enrolled in this study and divided into four groups based on quartiles of serum klotho concentrations. Patient characteristics are shown in Table 1. The proportion of male patients decreased with increasing klotho concentration quartiles, as did those who drank alcohol. The ratio of klotho concentrations varied among the study cycles. Other covariates were not significantly different among the four groups.

**Table 1:** The characteristics of RA patients according to the serum klotho concentration

Characteristics	Total	Serum klotho				
		**Q1**	**Q2**	**Q3**	**Q4**	***p* value**
	*N* = 841	*N* = 211	*N* = 209	*N* = 210	*N* = 211	
**Gender (%)**						0.024
Male	354 (42.09)	97 (45.97)	95 (45.45)	92 (43.81)	70 (33.18)	
Female	487 (57.91)	114 (54.03)	114 (54.55)	118 (56.19)	141 (66.82)	
**Age, years (%)**						0.358
< 60	355 (42.21)	86 (40.76)	86 (41.15)	83 (39.52)	100 (47.39)	
≥ 60	486 (57.79)	125 (59.24)	123 (58.85)	127 (60.48)	111 (52.61)	
**Race (%)**						0.170
Non-Hispanic White	332 (39.48)	82 (38.86)	92 (44.02)	92 (43.81)	66 (31.28)	
Non-Hispanic Black	251 (29.84)	64 (30.33)	56 (26.79)	53 (25.24)	78 (36.97)	
Mexican American	128 (15.22)	31 (14.69)	34 (16.27)	31 (14.76)	32 (15.17)	
Others	130 (15.46)	34 (16.11)	27 (12.92)	34 (16.19)	35 (16.59)	
**Education level (%)**						0.092
Less than 9th grade	130 (15.46)	34 (16.11)	30 (14.35)	30 (14.29)	36 (17.06)	
9–11th grade	155 (18.43)	52 (24.64)	37 (17.70)	29 (13.81)	37 (17.54)	
High school graduate	189 (22.47)	41 (19.43)	50 (23.92)	60 (28.57)	38 (18.01)	
Some college or AA degree	265 (31.51)	64 (30.33)	66 (31.58)	69 (32.86)	66 (31.28)	
College graduate or above	102 (12.13)	20 (9.48)	26 (12.44)	22 (10.48)	34 (16.11)	
**Marital status (%)**						0.044
Married or living with partner	480 (57.07)	120 (56.87)	134 (64.11)	124 (59.05)	102 (48.34)	
Widowed, divorced, or separated	293 (34.84)	73 (34.60)	60 (28.71)	74 (35.24)	86 (40.76)	
Never married	68 (8.09)	18 (8.53)	15 (7.18)	12 (5.71)	23 (10.90)	
**Family poverty income ratio (%)**						0.526
0.00–1.00	239 (28.42)	63 (29.86)	57 (27.27)	60 (28.57)	59 (27.96)	
1.00–3.00	375 (44.59)	103 (48.82)	90 (43.06)	88 (41.90)	94 (44.55)	
≥ 3.00	227 (26.99)	45 (21.33)	62 (29.67)	62 (29.52)	58 (27.49)	
**BMI, kg/m**^**2**^** (%)**						0.422
< 25.0	158 (18.79)	37 (17.54)	40 (19.14)	39 (18.57)	42 (19.91)	
25.0–30.0	238 (28.30)	58 (27.49)	66 (31.58)	66 (31.43)	48 (22.75)	
≥ 30.0	445 (52.91)	116 (54.98)	103 (49.28)	105 (50.00)	121 (57.35)	
**Ever drinking (%)**						0.054
Yes	566 (67.30)	153 (72.51)	142 (67.94)	144 (68.57)	127 (60.19)	
No	275 (32.70)	58 (27.49)	67 (32.06)	66 (31.43)	84 (39.81)	
**Cotinine, ng/ml**	0.07 (0.02,92.10)	0.13 (0.02,129.00)	0.09 (0.02,163.00)	0.07 (0.02,19.30)	0.05 (0.02,1.06)	0.303
**Hypertension (%)**						0.714
Yes	541 (64.33)	142 (67.30)	131 (62.68)	136 (64.76)	132 (62.56)	
No	300 (35.67)	69 (32.70)	78 (37.32)	74 (35.24)	79 (37.44)	
**Diabetes (%)**						0.661
Yes	239 (28.42)	54 (25.59)	58 (27.75)	62 (29.52)	65 (30.81)	
No	602 (71.58)	157 (74.41)	151 (72.25)	148 (70.48)	146 (69.19)	
**eGFR, mL/(min × 1.73 m**^**2**^**) (%)**						0.013
< 60.0	119 (14.15)	43 (20.38)	29 (13.88)	27 (12.86)	20 (9.48)	
≥ 60.0	722 (85.85)	168 (79.62)	180 (86.12)	183 (87.14)	191 (90.52)	
**Physical activity, MET time/week (%)**						0.976
< 600	446 (53.03)	114 (54.03)	106 (50.72)	113 (53.81)	113 (53.55)	
600–1200	87 (10.34)	22 (10.43)	20 (9.57)	23 (10.95)	22 (10.43)	
≥ 1200	308 (36.63)	75 (35.55)	83 (39.71)	74 (35.24)	76 (36.02)	
**25-hydroxyvitamin D, nmol/L (%)**						0.780
< 50.0	271 (32.22)	148 (70.14)	142 (67.94)	142 (67.62)	138 (65.40)	
≥ 50.0	570 (67.78)	63 (29.86)	67 (32.06)	68 (32.38)	73 (34.60)	
**NHANES cycles (%)**						0.006
2007–2008	199 (23.67)	55 (26.07)	42 (20.10)	53 (25.24)	49 (23.22)	
2009–2010	180 (21.40)	51 (24.17)	54 (25.84)	38 (18.10)	37 (17.54)	
2011–2012	143 (17.00)	24 (11.37)	33 (15.79)	47 (22.38)	39 (18.48)	
2013–2014	146 (17.36)	26 (12.32)	38 (18.18)	43 (20.48)	39 (18.48)	
2015–2016	173 (20.57)	55 (26.07)	42 (20.10)	29 (13.81)	47 (22.27)	

*BMI* body mass index, *CI* confidence interval, *eGFR* estimated glomerular filtration rate, *HR* hazard ratio, *PIR* poverty income ratio

### Serum klotho and mortality

**Fig. 2:**
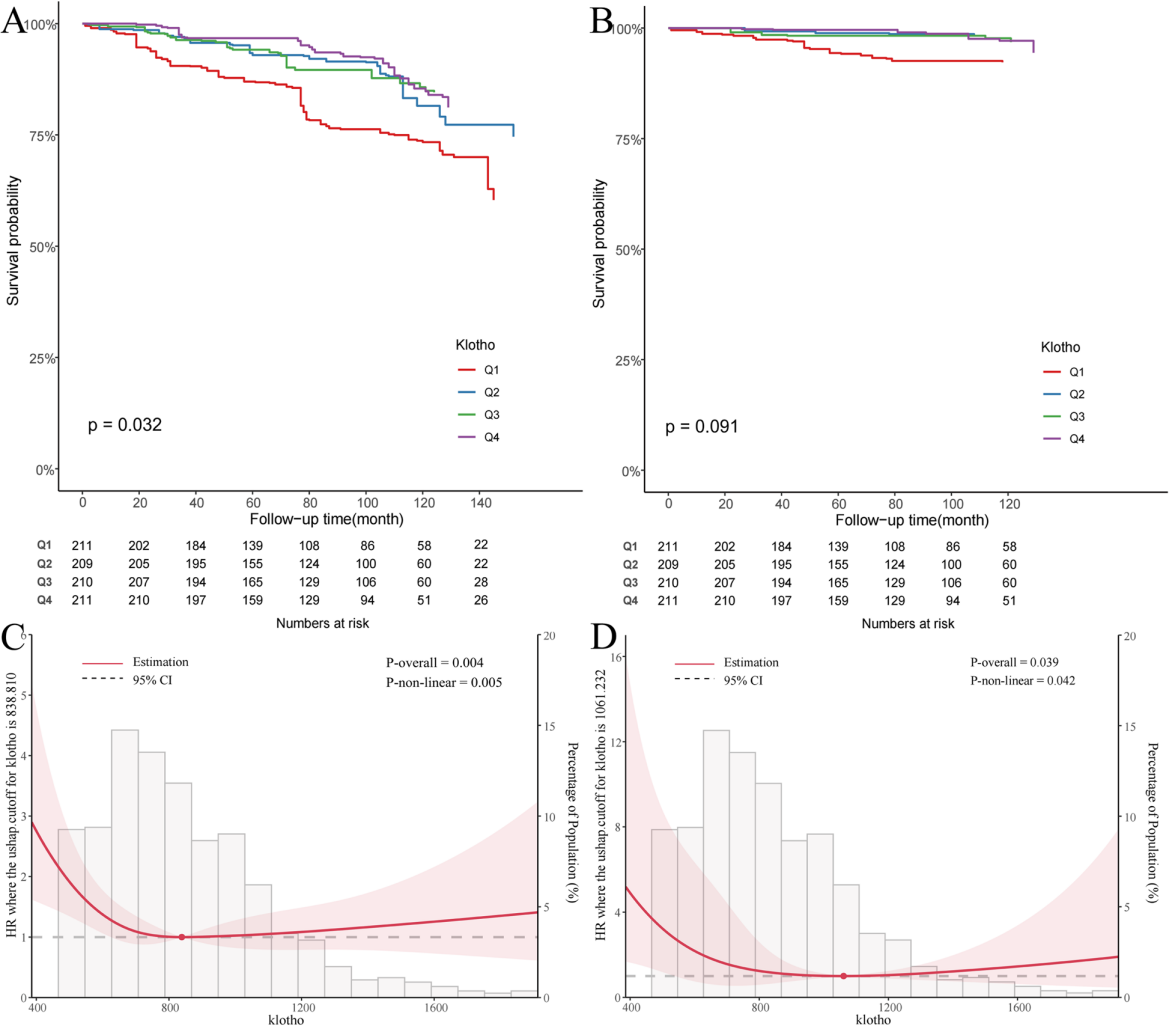
The association between serum klotho and mortality. The KM survival curve of the study populations based on the klotho group: **A** all-cause mortality and **B** cardiovascular mortality; the RCS with cox regression models on the klotho levels: **C** all-cause mortality and **D** cardiovascular mortality, adjusting for gender, age, race, education level, marital status, PIR, cotinine, drinking, hypertension, diabetes, BMI, physical activity, eGFR, 25-hydroxyvitamin D, and study cycle

There were 144 documented deaths among the 841 included patients; 35 patients died due to cardiovascular events. We established KM survival curves based on serum klotho concentrations and found a significantly lower overall survival rate in patients with low serum klotho levels compared to those with high serum klotho levels (Fig. 2A, B). After adjusting for multiple variables, including sex, age, race, education level, marital status, PIR, cotinine, drinking, hypertension, diabetes, BMI, physical activity, eGFR, 25-hydroxyvitamin D, and study cycle, we found that patients with high serum klotho levels (Q2, Q3, and Q4) had a lower risk of all-cause mortality than patients with low serum klotho levels (Q1) (*P* < 0.05) (Table 2). The results of the multivariate adjustment (model 3) suggested that serum klotho levels and the all-cause mortality rate were nonlinearly correlated. A similar trend and nonlinear correlation were indicated by the Cox regression analysis of the association between cardiovascular mortality and klotho concentrations.

**Table 2:** The HR (95% CIs) for all-cause and cardiovascular mortality according to serum Klotho concentration

	Serum klotho concentration (pg/ml)			
	Q1	Q2	Q3	Q4
All-cause mortality				
Number of deaths (%)	55 (26.07%)	29 (13.88%)	31 (14.76%)	29 (13.74%)
Model 1: HR (95% CI), *P* value	Ref	0.51 (0.26, 0.99) 0.049	0.45 (0.24, 0.81) 0.008	0.39 (0.22, 0.67) 0.001
Model 2: HR (95% CI), *P* value	Ref	0.49 (0.25, 0.97) 0.040	0.43 (0.23, 0.79) 0.007	0.43 (0.23, 0.70) 0.001
Model 3: HR (95% CI), *P* value	Ref	0.49 (0.25, 0.96) 0.038	0.44 (0.24, 0.81) 0.008	0.41 (0.24, 0.71) 0.001
Cardiovascular mortality				
Number of deaths	18 (8.53%)	4 (1.91%)	6 (2.86%)	7 (3.32%)
Model 1: HR (95% CI), *P* value	Ref	0.21 (0.06, 0.76) 0.010	0.32 (0.08, 1.23) 0.100	0.30 (0.10, 0.88) 0.030
Model 2: HR (95% CI), *P* value	Ref	0.18 (0.06, 0.58) 0.004	0.26 (0.07, 0.93) 0.039	0.30 (0.12, 0.76) 0.012
Model 3: HR (95% CI), *P* value	Ref	0.12 (0.04, 0.38) 0.001	0.27 (0.08, 0.91) 0.035	0.24 (0.10, 0.54) 0.001

Model 1 was non-adjusted; model 2 was adjusted for sex, age, race, and study cycle; model 3 was adjusted for sex, age, race, education level, marital status, PIR, cotinine, drinking, hypertension, diabetes, BMI, physical activity, eGFR, 25-hydroxyvitamin D, and study cycle

*BMI* body mass index, *CI* confidence interval, *eGFR* estimated glomerular filtration rate, *HR* hazard ratio, *PIR* poverty income ratio

Further investigation of the relationship between the serum klotho level and all-cause or cardiovascular mortality via Cox proportional hazard regression with RCS models demonstrated a U-shaped association. The tangent point of the U-shaped curve for all-cause mortality was 838.81 pg/mL. When the serum klotho concentration was less than 838.81 pg/mL, there was a significant downward trend in the risk of death as the klotho concentration increased. In contrast, there was a slight upward trend in the risk of death as the concentration increased above 838.81 pg/mL (Fig. 2C). Serum klotho exhibited a similar role in cardiovascular mortality, with the tangent point of the U-shaped curve detected at 1061.23 pg/mL (Fig. 2D).

### Subgroup analysis of all-cause mortality

**Fig. 3:**
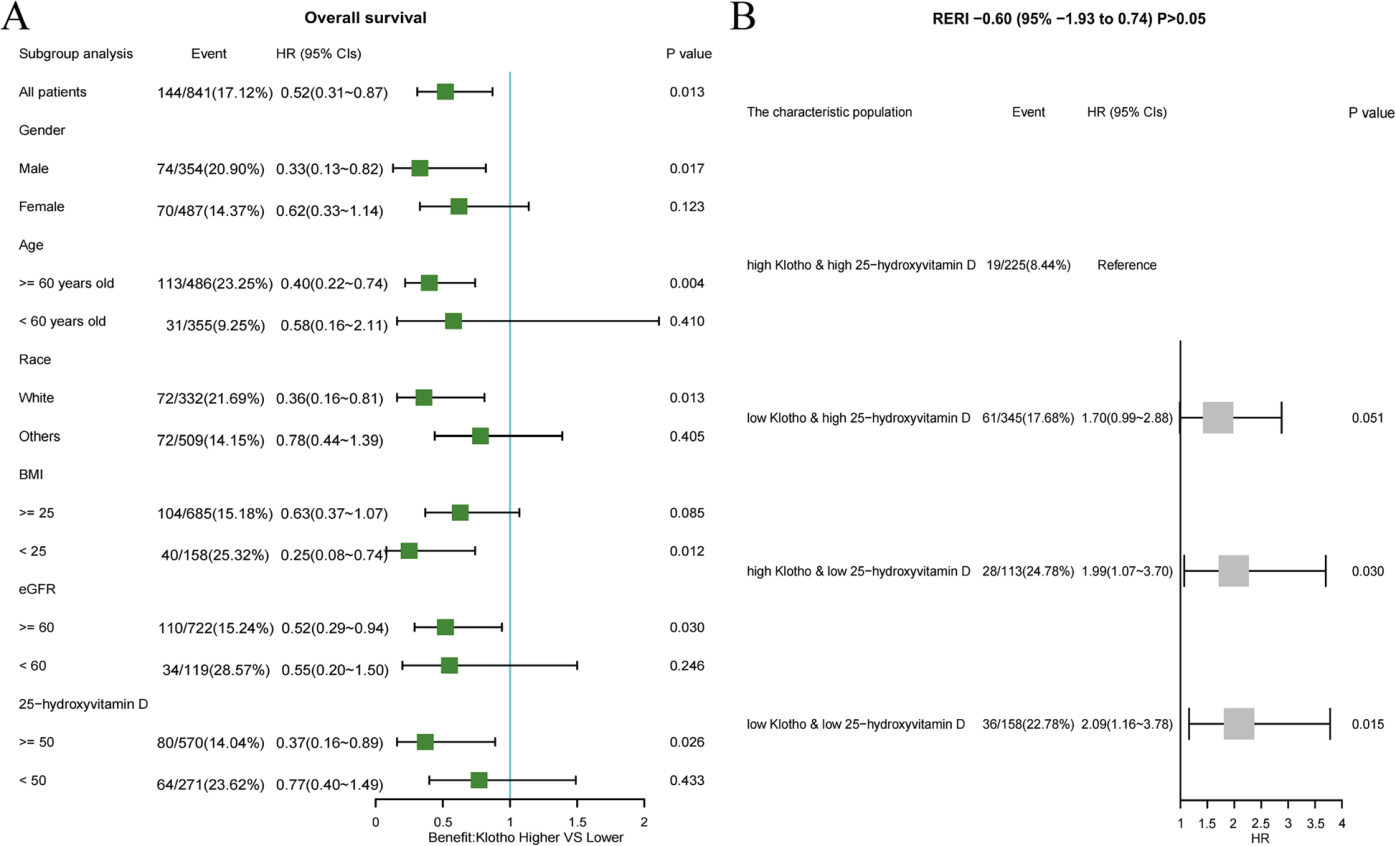
The forest map. **A** HRs of benefit with high klotho vs low klotho for all-cause mortality according to the subgroups (sex, age, ethnicity, BMI, eGFR and 25-hydroxyvitamin D). **B** The association of 25-hydroxyvitamin D with all-cause mortality by serum klotho

**Fig. 4:**
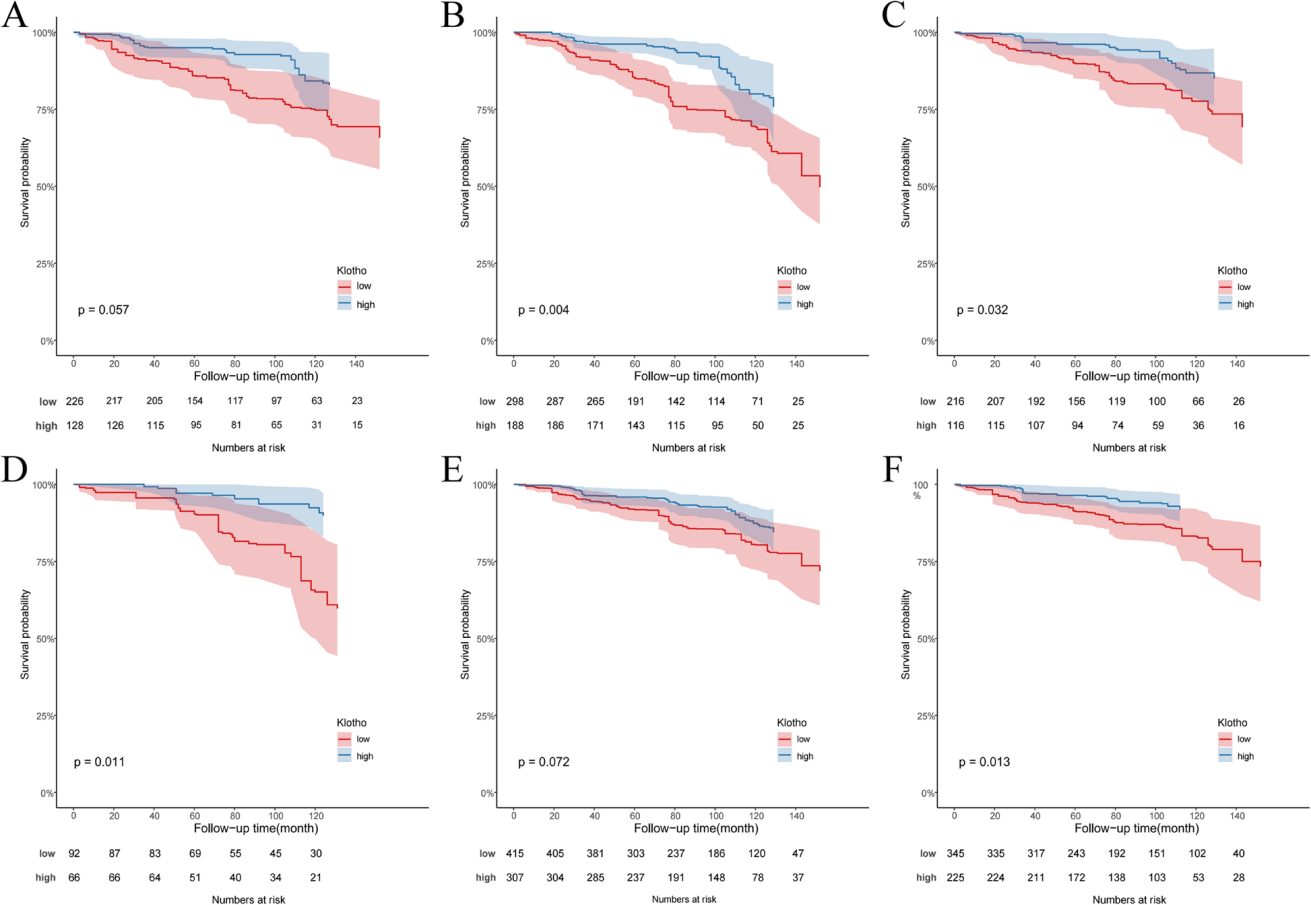
The KM survival curve of the study populations based on the subgroups. **A** male; **B** age ≥ 60 years old; **C** White; **D** BMI < 25 kg/m^2^; **E** eGFR ≥ 60 mL/(min × 1.73 m^2^); **F** 25-hydroxyvitamin D ≥ 50 nmol/L

Based on the aforementioned tangent point (838.81 pg/mL) of klotho for all-cause mortality, we further divided the population into a high serum klotho concentration group and a low serum klotho concentration group. Cox regression analysis showed that the high concentration group had a lower risk of all-cause mortality than the low concentration group (HR [95% CI]: 0.52 (0.31, 0.87), *P* = 0.02). Subgroup analysis based on sex (male or female), age (≥ 60 or < 60 years old), ethnicity (white or others), BMI (≥ 25 or < 25 kg/m^2^), eGFR (≥ 60 or < 60 mL/[min*1.73 m^2^]), and 25-hydroxyvitamin D (≥ 50 or < 50 nmol/L) found that high serum klotho concentrations were more beneficial in patients with the following characteristics: male, white ethnicity, age older than 60 years old, BMI < 25 kg/m2, eGFR ≥ 60 mL/ (min × 1.73 m^2^), and 25-hydroxyvitamin D ≥ 50 nmol/L (Fig. 3A). The KM survival curves for the subgroup analysis revealed that this effect was more pronounced in Caucasians older than 60 years, with a BMI of < 25 kg/m^2^ and 25-hydroxyvitamin D level of ≥ 50 nmol/L (Fig. 4).

As klotho exhibited a better protective effect in patients with high 25-hydroxyvitamin D levels, we attempted to determine whether there was an interaction between klotho and 25-hydroxyvitamin D. The multiplicative interaction suggested a lack of significant interaction (HR [95% CI]: 0.62 (0.30, 1.29), *P* = 0.19). Further additive interaction analysis revealed an increased risk of death in the high klotho and low 25-hydroxyvitamin D population (HR [95% CI]: 1.99 [1.07, 3.70], *P* = 0.03), as well as the low klotho and low 25-hydroxyvitamin D population (HR [95% CI]: 2.09 [1.16, 3.78], *P* = 0.01), compared to the high klotho and high 25-hydroxyvitamin D population. However, there was no overall significant difference (relative excess risk due to the interaction: − 0.60 [− 1.93, 0.74]) (Fig. 3B).

## Discussion

This was the first prospective study to evaluate the association between serum klotho concentrations and RA mortality by analyzing a cohort of RA patients from the NHANES 2007–2016. We found a U-shaped relationship between serum klotho concentrations and mortality, suggesting that low serum klotho concentrations were significantly associated with higher all-cause and cardiovascular mortality among patients with RA. These findings indicate that prompt identification and treatment of RA patients with low serum klotho concentrations may be necessary to prevent mortality.

From the U-shaped curve, we found that the tangent point for serum klotho was 838.81 pg/mL. When the klotho concentration was < 838.81 pg/mL, a further decrease klotho level was significantly associated with an increased risk of all-cause mortality (HR [95% CI]: 0.996 [0.994,0.998],* P* < 0.01); in contrast, there was a very slight increase in mortality when the klotho concentrations exceed 838.81 pg/mL, and the change almost leveled off (HR [95% CI]: 1.000 [0.999,1.001], *P* = 0.96). A subgroup analysis for all-cause mortality that was based on sex, age, ethnicity, BMI, eGFR, and 25-hydroxyvitamin D found that high serum klotho concentrations were more beneficial among RA patients who were white, older than 60 years, and with a BMI of < 25 kg/m2 and 25-hydroxyvitamin D level of ≥ 50 nmol/L.

In recent years, several studies have focused on the role of klotho in RA. Klotho may be involved in the regulation of T-cell senescence, as a lower expression of both mRNA and proteins has been observed in CD4^+^ cells of patients with RA compared to healthy controls; furthermore, klotho in patients with RA has been shown to be significantly associated with lower glucuronidase activity and negatively correlated with age [18]. Another study reported that klotho expression was significantly reduced in the CD4^+^ cells of patients with RA and that this was consistent with the downregulation of CD28 [19]. Klotho expression has also been detected in the synovial tissue; while expression was found to be significantly lower in synovial fibroblasts than in the synovial tissue of origin, the exact mechanism is unclear [20]. Acute aerobic exercise in patients with RA may exert an anti-inflammatory effect by upregulating klotho [21]. Serum klotho levels were found to be higher in patients with RA than in healthy controls and were positively correlated with anti-citrullinated peptide antibodies and rheumatoid factor; the levels were higher in the biologic treatment group than in the conventional treatment group [15]. These results suggest that klotho plays a significant negative regulatory role in RA. However, its exact mechanism of action and effect on patient mortality remain unclear.

Disease activity is one of the most important factors in assessing the efficacy and prognosis of RA, but studies on the association between klotho and disease activity are still limited. Alvarez-Cienfuegos and his colleagues found a negative correlation between plasma klotho levels and disease activity (DAS28-ESR) (correlation coefficient =  − 0.22, *p* = 0.07) in the included 63 female RA patients, but no significant correlation between FGF23, an important ligand for klotho, and disease activity (correlation coefficient = 0.08, *p* = 0.52) [15]. Another study that included 61 female RA patients found a positive correlation between FGF23 and DAS28-ESR (correlation coefficient = 0.41, *p* < 0.01) [22]. The differences in age, BMI, disease activity, and treatment regimens in the study populations included in the two studies may account for the inconsistent results. We were unable to further explore the relationship between klotho and disease activity due to the lack of counts of pain and swelling joint in RA patients in the NHANES database. The relationship between serum klotho and disease activity and prognosis still needs further validation in clinical cohorts.

Several studies have confirmed an association between low serum klotho levels and mortality in specific diseases. Controversy remains regarding the association between serum klotho levels and outcomes in patients undergoing hemodialysis. A single-center cohort study found no significant correlation between serum klotho concentrations and the risk of death in hemodialysis patients [23]. A negative association was observed between soluble klotho and all-cause and cardiovascular mortality in patients undergoing maintenance hemodialysis [24]. Single-nucleotide polymorphisms in the klotho gene may help predict non-cardiovascular mortality in patients with chronic kidney disease [25]. An analysis of a cohort from the NHANES found that lower klotho concentrations in people with hypertension were associated with higher all-cause mortality, but not cardiovascular mortality [26]. Low serum klotho levels were reported to be negatively associated with all-cause mortality in people older than 40 years in the USA [27]. Another study suggested that serum klotho levels were negatively associated with cardiovascular mortality, but not associated with all-cause mortality, in hemodialysis patients with no or mild abdominal aortic calcification [28]. A prospective cohort study reported an independent association between elevated serum klotho levels and higher mortality in patients with septic shock [29]. Our study is the first to suggest that serum klotho is strongly associated with all-cause mortality in RA.

Studies conducted in recent years have greatly advanced the understanding of the role and mechanism of klotho in a wide range of diseases [14, 30–32], particularly those involving the kidney. The protective effects of klotho primarily comprise anti-oxidation, induction of autophagy, and the inhibition of cellular senescence and apoptosis. Klotho can inhibit cellular senescence by regulating expression of the Wnt signaling pathway [33], and inflammatory factors such as interleukin-6 (IL-6) [34]. Klotho may also be involved in the regulation of oxidative stress injury and apoptosis in kidney cells [35]. A prior study using a mouse model of immune-mediated glomerulonephritis reported that klotho attenuated renal injury by reducing mitochondrial DNA fragmentation, superoxide anion production, lipid peroxidation, and apoptosis in kidney tissues [36]. By upregulating autophagy, klotho can reduce ischemic kidney injury and mitigate progression to chronic kidney disease [37]. Klotho is also involved in maintaining the integrity of the vascular endothelium [38] and inhibiting fibrosis in the kidneys [39]. Therefore, we hypothesized that the soluble klotho could inhibit systemic and local synovial inflammation in RA patients by suppressing the production of inflammatory factors such as tumor necrosis factor and IL-6, and participate in the regulation of local synovial cell proliferation and pannus to improve disease activity and prognosis. And the specific mechanism of action of klotho in RA requires more in-depth experimental validation.

Additionally, klotho has been reported to participate in the regulation of vitamin D [40]. Elevated circulating vitamin D levels promote the expression of FGF23 in the bone; FGF23 subsequently enters the circulation and partially binds to klotho to regulate vitamin D expression [41]. Klotho is also involved in the regulation of calcium and phosphorus metabolism [42] and the expression of parathyroid hormones [43]. Our study found that klotho had a greater protective effect in patients with high levels of vitamin D. Further analysis of multiplicative and additive interactions did not reveal a significant interaction effect between klotho and vitamin D; a larger cohort may be required for further validation.

The stimulation of exogenous or endogenous klotho expression to achieve anti-aging and anti-inflammatory effects may have potential as a new therapeutic strategy; however, to date, there have been no human studies on the klotho protein. Nevertheless, klotho treatment has been reported in several animal models. Preclinical data suggest that exogenous soluble klotho-based proteins have therapeutic potential for age-related diseases and diseases associated with klotho deficiency [44]. The klotho automatic production model made of special materials was better confirmed in mouse models of ischemia–reperfusion injury and unilateral ureteral obstruction [45]. In addition, some drugs such as vitamin D receptor agonists [40], sevelamer carbonates [46], and statins [47] can elevate the expression of klotho. There are no clinically applicable specific agonists for klotho, and the application of klotho in RA needs to be explored and validated in depth.

While our use of Cox regression analysis, KM curve analysis, and the RCS model was able to indicate the importance of klotho levels in patients with RA, our study has some limitations. First, owing to the lack of repeated klotho measurements, we could not determine the association between dynamic klotho levels and mortality. Second, since klotho measurement in the NHANES was limited to patients aged ≥ 40 years, our results may not be generalizable to patients with RA in other age groups. Third, the definition of RA was based on the following question: “which type of arthritis was it?” This may have biased the results to some extent, as we were unable to further confirm the applicable diagnostic criteria. Fourth, the low prevalence of cardiovascular mortality in the cohort may have influenced the statistical analysis of its association with serum klotho levels. Last but not the least, while we adjusted for some covariates in the analysis, we could not exclude the possibility that other covariates, which were not included, affected mortality or klotho levels. Therefore, large prospective cohort studies evaluating dynamic klotho levels are required to validate the correlation between serum klotho levels and RA mortality.

## Conclusions

In conclusion, our study analyzed the association of serum klotho levels with both all-cause and cardiovascular mortality in patients with RA using NHANES data. We observed a significant U-shaped association between serum klotho levels and all-cause mortality, which suggests that lower serum klotho was significantly associated with higher all-cause mortality in RA patients, especially those serum klotho < 838.81 pg/mL. Our results indicate a protective role of klotho on patients with RA, which provides a theoretical basis for subsequent experimental validation, as well as new insights into potential interventions for improving survival with adequate serum klotho in this patient group.

## Data Availability

The data underlying this article are available in NHANES (https://www.cdc.gov/nchs/nhanes/).

## Abbreviations

BMI: Body mass index
CI: Confidence interval
eGFR: Estimated glomerular filtration rate
ELISA: Enzyme-linked immunosorbent assay
HR: Hazard ratio
KM: Kaplan–Meier
NHANES: National Health and Nutrition Examination Survey
PIR: Poverty income ratio
RCS: Restricted cubic spline
RA: Rheumatoid arthritis
